# Anthropogenic Zinc Exposure Increases Mortality and Antioxidant Gene Expression in Monarch Butterflies with Low Access to Dietary Macronutrients

**DOI:** 10.1002/etc.5305

**Published:** 2022-03-14

**Authors:** Alexander M. Shephard, Noah S. Brown, Emilie C. Snell‐Rood

**Affiliations:** ^1^ Department of Ecology, Evolution, and Behavior University of Minnesota Saint Paul Minnesota USA

**Keywords:** Heavy metal, Zinc, Nutritional ecology, Macronutrient, Monarch butterfly, Oxidative stress, Antioxidant

## Abstract

Biologists seek to understand why organisms vary in their abilities to tolerate anthropogenic contaminants, such as heavy metals. However, few studies have considered how tolerance may be affected by condition‐moderating factors such as dietary resource availability. For instance, the availability of crucial limiting macronutrients, such as nitrogen and phosphorous, can vary across space and time either naturally or due to anthropogenic nutrient inputs (e.g., agricultural fertilizers or vehicle emissions). Organisms developing in more macronutrient‐rich environments should be of higher overall condition, displaying a greater ability to tolerate metal contaminants. In monarch butterflies (*Danaus plexippus*), we factorially manipulated dietary macronutrient availability and exposure to zinc, a common metal contaminant in urban habitats that can be toxic but also has nutritional properties. We tested whether (1) the ability to survive zinc exposure depends on dietary macronutrient availability and (2) whether individuals exposed to elevated zinc levels display higher expression of antioxidant genes, given the roles of antioxidants in combatting metal‐induced oxidative stress. Exposure to elevated zinc reduced survival only for monarchs developing on a low‐macronutrient diet. However, for monarchs developing on a high‐macronutrient diet, elevated zinc exposure tended to increase survival. In addition, monarchs exposed to elevated zinc displayed higher expression of antioxidant genes when developing on the low‐macronutrient diet but lower expression when developing on the high‐macronutrient diet. Altogether, our study shows that organismal survival and oxidative stress responses to anthropogenic zinc contamination depend on the availability of macronutrient resources in the developmental environment. In addition, our results suggest the hypothesis that whether zinc acts as a toxicant or a nutrient may depend on macronutrient supply. *Environ Toxicol Chem* 2022;41:1286–1296. © 2022 The Authors. *Environmental Toxicology and Chemistry* published by Wiley Periodicals LLC on behalf of SETAC.

## INTRODUCTION

Anthropogenic activities expose natural populations to a range of chemical contaminants, including pesticides, pharmaceuticals, and heavy metals (Birch et al., 2015). Of these, heavy metals are among the most common and widespread contaminants associated with human activity (Monchanin et al., 2021), released from sources such as vehicle wear‐and‐tear, railroads, industrial combustion, and mining (Lagerwerff & Specht, 1970; Wolz et al., 2003). Although previous research has demonstrated that individuals, populations, and species can vary remarkably with respect to metal tolerance (Antonovics, 1970; Harper et al., 1997; Levinton et al., 2003; Morgan et al., 2007; Posthuma & Van Straalen, 1993; Shephard et al., 2021), experimental approaches rarely account for condition‐moderating factors that might influence pollution tolerance under ecologically relevant settings (e.g., competition, nutrient limitation, temperature fluctuations, or predation). Given the potential physiological costs of heavy metal tolerance (Morgan et al., 2007; Posthuma & Van Straalen, 1993), lower condition individuals should be less tolerant than higher condition individuals.

The quality or availability of dietary resources is a central driver of organismal condition that may influence tolerance to pollutants such as heavy metals. Across diverse taxa, organismal condition is moderated by natural variation in resource availability, from temporal changes in prey availability for predators (Lack, 1954; Poulin et al., 1992) to variation in food plant quality for insect herbivores (Scriber & Slansky, 1981). Differences in organismal condition, as moderated by nutrient or resource availability, have long been hypothesized to explain variation in life‐history strategies (Bonduriansky, 2007; Cody, 1966; Houle, 1991; van Noordwijk & de Jong, 1986; Zera & Harshman, 2001): genotypes with a greater ability to acquire or assimilate resources can afford to invest more in various fitness‐related traits (e.g., growth rate, fecundity, body size, sexual signaling) relative to more resource‐limited individuals. In particular, the availability of key dietary macronutrients (e.g., nitrogen and phosphorous) might have a key influence on variation in organismal condition, given their important roles in metabolic function and development. Indeed, fitness‐related traits such as growth and fecundity are often limited by macronutrient availability (Jeyasingh & Weider, 2005; Morehouse & Rutowski, 2010; Sterner, 1993). However, apart from a few examples in the ecotoxicology literature (see Peace et al., 2021), few studies have examined the interactive effects of anthropogenic pollutants and nutrient availability on organismal performance or the underlying mechanisms that may drive such interactions.

Nutrient‐dependent tolerance to heavy metal pollution should be influenced by underlying physiological responses. For instance, oxidative stress responses are a well‐known consequence of heavy metal exposure. Some heavy metals (e.g., copper and iron) generate oxidative stress by removing electrons from water molecules, producing reactive oxygen species (ROS) such as superoxide radicals, hydroxyl radicals, and hydrogen peroxide (Ercal et al., 2001). Other metals, including lead, cadmium, zinc, and mercury, generate ROS indirectly by first interfering with cellular functions, such as enzyme activity, cell membrane integrity, or mitochondrial performance (Capasso et al., 2005; Ercal et al., 2001; Gazaryan et al., 2007). If not mitigated, ROS can damage DNA, lipids, and proteins (Koch & Hill, 2017). In response to ROS challenge, organisms up‐regulate a range of defenses, including antioxidant pathways, in an attempt to mitigate oxidative stress (Costantini et al., 2010; Riley, 1994). Indeed, organisms often respond to heavy metal exposure by increasing antioxidant expression (see Liu et al., 2018; Somasundaram et al., 2019; Zheng et al., 2016). Some evidence indicates that more resource‐limited individuals experience higher levels of oxidative stress (Fletcher et al., 2013; Tökölyi et al., 2014), suggesting that the ability to mitigate oxidative stress may be nutrient or resource dependent. However, it remains unclear how variation in nutrient availability and exposure to pollutants such as heavy metals may interact to affect oxidative stress responses.

In the present study, we focused on larvae of the monarch butterfly (*Danaus plexippus*) to test how variation in dietary macronutrient availability influences heavy metal tolerance. Heavy metals are a persistent threat to insect pollinators such as monarchs, because metal contamination can occur in host plant leaf tissue and nectar in polluted areas (Monchanin et al., 2021; Shephard et al., 2020). We specifically focused on the impacts of dietary zinc contamination. Although zinc serves as an essential micronutrient at trace doses, it is toxic at higher levels, generating ROS by interfering with biochemical processes involved in energy production and enzyme activity (Capasso et al., 2005; Gazaryan et al., 2007). Zinc is commonly released from agricultural runoff or vehicle brake pad wear‐and‐tear (Jaradat & Momani, 1999; Lagerwerff & Specht, 1970) and can accumulate in soils, where it may transfer into plant leaf tissue and be consumed by caterpillars (Mitchell et al., 2020). Metal pollution and nutrient availability may often covary in the diets of insect pollinators in human‐impacted areas, causing insects to be exposed to metal pollution in the context of both low‐ and high‐quality plants. For instance, along roadsides, metals and other pollutants (e.g., road de‐icing salts) can cause stress to pollinator host plants and significantly lower their nutritional quality (Hu & Schmidhalter, 2005). At the same time, plant nutrient availability is often increased by anthropogenic macronutrient outputs, such as nitrogen and phosphorous from agriculture and road traffic (Vitousek et al., 1997). Vehicle emissions, in particular, are the largest source of anthropogenic nitrogen release in the United States (Bettez et al., 2013).

By testing the interactive effects of dietary macronutrient availability and zinc exposure on monarch performance, we explored the hypothesis that heavy metal tolerance is dependent on dietary nutrient availability. In a rearing experiment, we exposed larvae of wild‐caught monarch butterflies to varying levels of zinc exposure and dietary macronutrient availability. Given the intimate link between heavy metal exposure and oxidative stress responses (Koch & Hill, 2017), we also quantified expression of a suite of antioxidant genes in monarch larvae (Table 1). Because more nutrient‐limited individuals should face higher costs of metal tolerance (Posthuma & Van Straalen, 1993), we predicted that monarchs developing on the low‐macronutrient diet would display lower survival, prolonged development time, slower growth rate, smaller adult body size, and reduced egg production when exposed to elevated zinc exposure. In invertebrates, antioxidant defenses are commonly induced in response to oxidative stress burdens (De Block & Stoks, 2008; Krishnan & Kodrík, 2006; Mittapalli et al., 2007), including those induced by zinc exposure (Geret & Bebianno, 2004; Liu et al., 2018; Somasundaram et al., 2019; Trevisan et al., 2014; Wilczek et al., 2004). Thus, we predicted that monarchs exposed to elevated zinc would exhibit increased expression of antioxidant genes. Given that this oxidative stress burden should be even greater for more nutrient‐limited individuals, we also predicted that relative to monarchs on the high‐macronutrient diet, monarchs on the low‐macronutrient diet would have higher levels of antioxidant gene expression after exposure to zinc.

**Table 1:** Summary of candidate antioxidant genes measured in monarch butterfly larvae (*Danaus plexippus*)

Gene	Protein	Role in oxidative stress or heavy metal regulation
*SOD1*	Superoxide dismutase	First line of defense against ROS: converts superoxide into hydrogen peroxide (Sagara et al., 1998)
*CAT*	Catalase	Converts hydrogen peroxide into oxygen and water (Sagara et al., 1998)
*PRDX1*	Peroxiredoxin	Converts hydrogen peroxide into water (Sagara et al., 1998)
*TSA1*	Thioredoxin peroxidase	Involved in conversion of hydrogen peroxide to water (Sagara et al., 1998)
*FER1HCH*	Ferritin	Transport and storage of metals, especially iron and zinc (Price & Joshi, 1982)
*TSF1*	Transferrin	Regulates levels of free iron (Price & Joshi, 1982)
*GSTD1*	Glutathione s‐transferase delta 1	Involved in antioxidant responses and detoxification (Corona & Robinson, 2006)
*GSTO3*	Glutathione s‐transferase omega 3	Involved in antioxidant responses to heat and heavy metal stress, particularly in insects (Lee et al., 2015)
*TH*	Tyrosine hydroxylase	Initial enzyme in melanin pathway; catalyzes conversion of tyrosine into L‐DOPA (Nagatsu, 1995)

## MATERIALS AND METHODS

### Origin of butterflies

All experimental monarch larvae originated from eggs produced by more than 10 wild‐collected females reared in common garden conditions. These parental individuals were originally collected as eggs or early instar larvae from the University of Minnesota Saint Paul (USA) campus over a 3‐week period in June 2019. To start a population for our rearing experiment, we reared wild‐caught individuals in the greenhouse in cages (60 × 60 × 60 cm) with ad libitum access to campus‐collected milkweed stalks (*Asclepias syriaca*) provided in floral wicks. We avoided collecting milkweed plants along roadsides. At eclosion, we transferred butterflies to large mating cages (24 × 24 × 36″) for 5–7 days prior to collecting eggs for the experiment (with five to six females and four to five males/mating cage). Butterflies in mating and egg collection cages had ad libitum access to 10% honey water provided in sponges. After mating, individual females were transferred to egg‐laying cages, each containing a single *A. syriaca* plant in a water wick and a moist towel to maintain humidity. We replaced plants daily. Each day, we transferred plants containing eggs to 32‐oz. plastic cups and stored the cups in a climate chamber (25 °C, 15:9‐h light:dark photoperiod). Eggs used for the experiment were collected from the parental females over 1 week in July 2019.

### Larval rearing

We reared monarch caterpillars on a semi‐artificial diet designed for monarchs by O.R Taylor at Monarch Watch, validated in previous studies (see Markert et al., 2016; Merlin et al., 2013). This approach allowed us to precisely control zinc exposure concentrations while simulating variation in host plant nutrient availability by diluting the nutritional component of the diet. The nutritional component of the diet consisted primarily of macronutrient sources including protein, lipids, and carbohydrates. Dried milkweed powder made up 15% of the diet. We reared monarch larvae in a 2 × 2 factorial experiment consisting of two factors (zinc exposure and macronutrient availability), with each factor containing two levels (control or elevated zinc exposure and high‐ or low‐macronutrient content). To prepare the elevated zinc treatment, we added 162 µl of 1 M ZnCl_2_ solution to the diet before blending. Zinc concentrations of the resulting diets were measured by dry weight using inductively coupled plasma‐atomic emission spectroscopy at the University of Minnesota Research Analytical Laboratory (St. Paul, MN, USA). On average, the zinc concentration of the elevated zinc diet was approximately 5× higher than the control diet (*t*‐test: *t*_5_ = 15.23, *SE* = 9.97, *p* < 0.0001; control zinc diet mean = 37.996 mg/kg, SD = 4.78, *n* = 3; elevated zinc diet mean = 189.77, SD = 16.41, *n* = 4). Our elevated zinc treatment was approximately in the range of zinc concentrations observed in plants along highways (~30–180 mg/kg; Jaradat & Momani, 1999; Lagerwerff & Specht, 1970) and near heavy metal smelters (~150–2000 mg/kg; Buchauer, 1973). For the low‐macronutrient diet, we removed 10.2 g (12%) of the dry nutrient component of the diet mix and replaced it with an equal mass portion of cellulose before blending. On average, the low‐macronutrient diet contained approximately 23% less phosphorous than the high‐macronutrient diet (*t*‐test: *t*_6_ = 18.87, SE = 79.15, *p* < 0.0001; low‐macronutrient diet mean = 6698.0 mg/kg, SD = 121.16, *n* = 4; high‐macronutrient diet mean = 8270.8, SD = 101.90, *n* = 4), and the low‐macronutrient diet contained approximately 25% less nitrogen than the high‐macronutrient diet (*t*‐test: *t*_6_ = 113.88, SE = 0.017, *p* < 0.001; low‐macronutrient diet mean = 5.875% nitrogen, SD = 0.021, *n* = 4; high‐macronutrient diet mean = 7.778% nitrogen, SD = 0.026, *n* = 4). The average carbon‐to‐nitrogen ratio of the low‐macronutrient diet was 7.731 (SD = 0.031, *n* = 4), and for the high‐macronutrient diet it was 5.885 (SD = 0.029, *n* = 4; *t*‐test: *t*_6_ = 87.66, SE = 0.021, *p* = <0.001). We did not manipulate the milkweed powder portion of the diet, because this serves as a feeding stimulus rather than a nutrient source.

After egg collection (at 5–6 days, second instar), we transferred larvae with a paintbrush from milkweed to one of the four treatments (40 larvae/treatment). Diet was provided in 16‐oz. plastic cups (2 larvae/cup). Throughout larval and pupal development, we housed all individuals in a climate chamber maintained at 25 °C with a 15:9‐h light:dark photoperiod. We positioned all cups on their sides to provide adequate space for butterfly eclosion. To confirm variation in monarch nutrient accumulation across treatments, thorax concentrations of zinc and phosphorous (in mg/kg) were measured in *n* = 6 adult butterflies from each treatment using inductively coupled plasma–mass spectrometry performed at the Quantitative Bio‐element Imaging Center, Northwestern University (Evanston, IL, USA). As expected, monarchs that developed on the elevated zinc diet tended to have higher thorax zinc concentrations, and monarchs developing on the high‐macronutrient diet tended to have higher thorax phosphorous concentrations (Supporting Information, Table S1 and Figure S1).

### Phenotypic performance measurements

We calculated monarch development time as the time between the day larvae were transferred to semi‐artificial diet treatment and the day of eclosion. Monarch survival was quantified as whether an individual survived to eclosion. After eclosion, we labeled all butterflies on the hindwing with a fine‐tipped black sharpie. All females that eclosed with undamaged wings were transferred to a large cage (61 × 61 × 61 cm) in the greenhouse for an egg development period of 7 days before being stored in a −20 °C freezer until ovary dissections. We measured egg production as the number of fully yoked and chlorinated eggs in the ovaries of dissected females. We also measured the average egg area (mm^2^) of five randomly selected mature eggs from each dissected female. All dissections were performed using a Leica M165C microscope at ×10 magnification while the butterfly abdomen was submerged in a 1× phosphate‐buffered saline buffer; eggs removed from the ovaries consistently lay on their sides in these conditions, facilitating an area measurement despite the three‐dimensional bullet shape of the egg.

We measured forewing length, a standard measure of adult body size in butterflies (Boggs, 1981; Gage, 1994), as the distance between the forewing apex and the articulation with the thorax. One forewing from each individual was carefully removed, laid flat, and photographed using a 50‐mm macrolens fitted to a Canon Rebel T3 camera. We measured forewing lengths using ImageJ (NIH). Growth rate was calculated as forewing length divided by development time.

### Gene selection

For primer development and gene expression analysis, we selected a set of candidate genes that are key components of antioxidant pathways conserved across animals (Halliwell & Gutteridge, 1999). We retrieved the sequences for all candidate genes from MonarchBase (Zhan & Reppert, 2013). We focused on a set of nine antioxidant genes that have well‐characterized roles in combating heavy metal stress in animals and fall into four categories (Table 1): neutralization of ROS, heavy metal sequestration, glutathione‐based detoxification, and production of melanin, a molecule that binds ROS to protect against oxidative stress (Sichel et al., 1991).

We initially selected two control genes for quantitative polymerase chain reaction (qPCR) analyses. First, we selected actin because it is one of the most frequently used reference genes for qPCR in insects and has been shown to be the most stably expressed reference gene across different developmental stages in a broad range of insect taxa (Pan et al., 2015), including Lepidoptera (Lu et al., 2013; Shu et al., 2018; Sikkink et al., 2020; Zhu et al., 2014). Second, we selected elongation factor 1‐alpha because this was shown by a recent study to be a stable reference gene in monarchs under a range of different biotic and abiotic conditions (Pan et al., 2015). However, in the present study we found that actin was stably expressed across treatment groups, but elongation factor 1‐alpha expression varied significantly across treatments (Supporting Information, Table S2). Therefore, only actin was used as a control gene for our analyses.

### RNA extraction and gene expression measurements

For gene expression analyses, we euthanized five monarch larvae from each of the four treatment combinations (control zinc/high‐macronutrient diet, elevated zinc/high‐macronutrient diet, control zinc/low‐macronutrient diet, and elevated zinc/low‐macronutrient diet). We extracted RNA from fat body tissue dissected from larvae at the fifth instar (17 days after transfer to each dietary treatment). Although there was no way to account for potential differences in development time across individuals at the time of dissection, analysis of variance indicated that there were no significant effects of macronutrient availability (*F*_1,__16_ = 2.33, *p* = 0.15), zinc exposure (*F*_1,__16_ = 0.0093, *p* = 0.92), or their interaction (*F*_1,__16_ = 0.47, *p* = 0.50) on caterpillar body mass prior to dissection. We dissected fat body tissue because this organ plays a central role in insect metabolic functions that are highly sensitive to variation in nutrient supply (Arreie & Soulages, 2010). In Lepidoptera larvae, increased expression of antioxidant genes in the fat body occurs in response to zinc‐induced oxidative stress (Liu et al., 2018). To enhance repeatability across individual samples, we removed fat from the second and sixth abdominal segments of each larva using forceps. We extracted RNA from fat body tissue using a QIAGEN RNeasy Micro kit (catalog no. 74004) under RNase‐free conditions. Harvested tissue was manually ground and placed in lysis buffer before flash freezing. We used RNA extracts from all 20 larvae for subsequent qPCR analysis because they all met the minimum yield requirements (minimum RNA extract sample yield = 809.4 ng/µl, maximum = 4987.9 ng/µl, mean = 2809.5 ng/µl).

We sent RNA extracts from all monarch larvae to the University of Minnesota Genomics Center (UMGC; Minneapolis, MN, USA) for complementary DNA synthesis using Invitrogen SuperScript II reverse transcriptase and quantitative real‐time (qRT‐)PCR. All PCR primers were developed and validated at UMGC; all qRT‐PCR was performed using an Applied Biosystems 7900HT real‐time PCR instrument. Two technical qPCR replicates were produced for each monarch RNA sample.

### Statistical analyses

For statistical analyses, we used R Studio Ver 3.6.3 (R Studio Team, 2020). We analyzed adult body size, development time, growth rate, egg number, and egg size using linear models with macronutrient availability, zinc exposure, sex, and the interaction between the macronutrient availability and zinc exposure as fixed effects. We analyzed survival to eclosion using a generalized linear model with a binomial distribution and a logit link function with macronutrient availability, zinc exposure, and the interaction between the two as fixed effects.

For each antioxidant gene, we analyzed effects of dietary treatment on expression using linear mixed effects models in the lme4 package (Bates et al., 2015). In each model, macronutrient availability, zinc exposure, and the interaction between the two were included as fixed effects. We included actin expression (normalizing gene) as a fixed effect in each model to control for the amount of starting template used in the reaction (Pfaffl, 2001). Technical qPCR replicate was included as a random effect. To facilitate interpretation, we multiplied all *C*_*t*_ values for gene expression by −1 prior to analyses so that higher values correspond to increased expression.

## RESULTS

### Effects of zinc exposure and macronutrient availability on monarch performance

**Figure 1:**
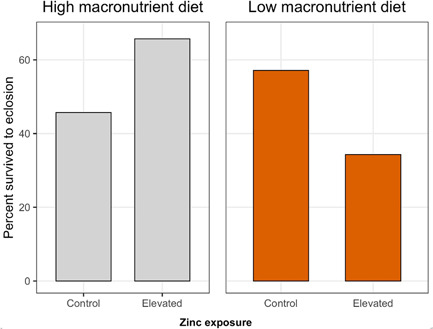
Effects of larval dietary zinc exposure and macronutrient availability on proportion survived from larva to adult eclosion in monarchs (*Danaus plexippus*). Each of the four treatment combinations contained *n* = 35 larvae.

Elevated zinc exposure tended to increase survival of monarchs developing on the high‐macronutrient diet (i.e., by ~20%; Figure 1), although this was not statistically significant (Table 2). In the absence of elevated zinc exposure, monarchs developing on the low‐macronutrient diet had approximately 15% higher survival than monarchs developing on the high‐macronutrient diet (Figure 1), but this was not statistically significant (Table 2). Elevated zinc exposure significantly reduced survival for monarchs developing on the low‐macronutrient diet by approximately 23% (Table 2 and Figure 1).

**Table 2:** Generalized linear model results for effects of larval macronutrient availability, zinc exposure, and the interaction between macronutrient availability and zinc exposure on survival from larva to eclosion in the monarch (*Danaus plexippus*)

	Estimate	SE	*z*	*p*
(Intercept)	−0.17	0.34	−0.51	0.61
Low‐macronutrient availability	0.46	0.48	0.95	0.34
Elevated zinc	0.82	0.49	1.67	0.094
Low‐macronutrient availability × Elevated zinc	−1.76	0.70	−2.53	0.011

**Figure 2:**
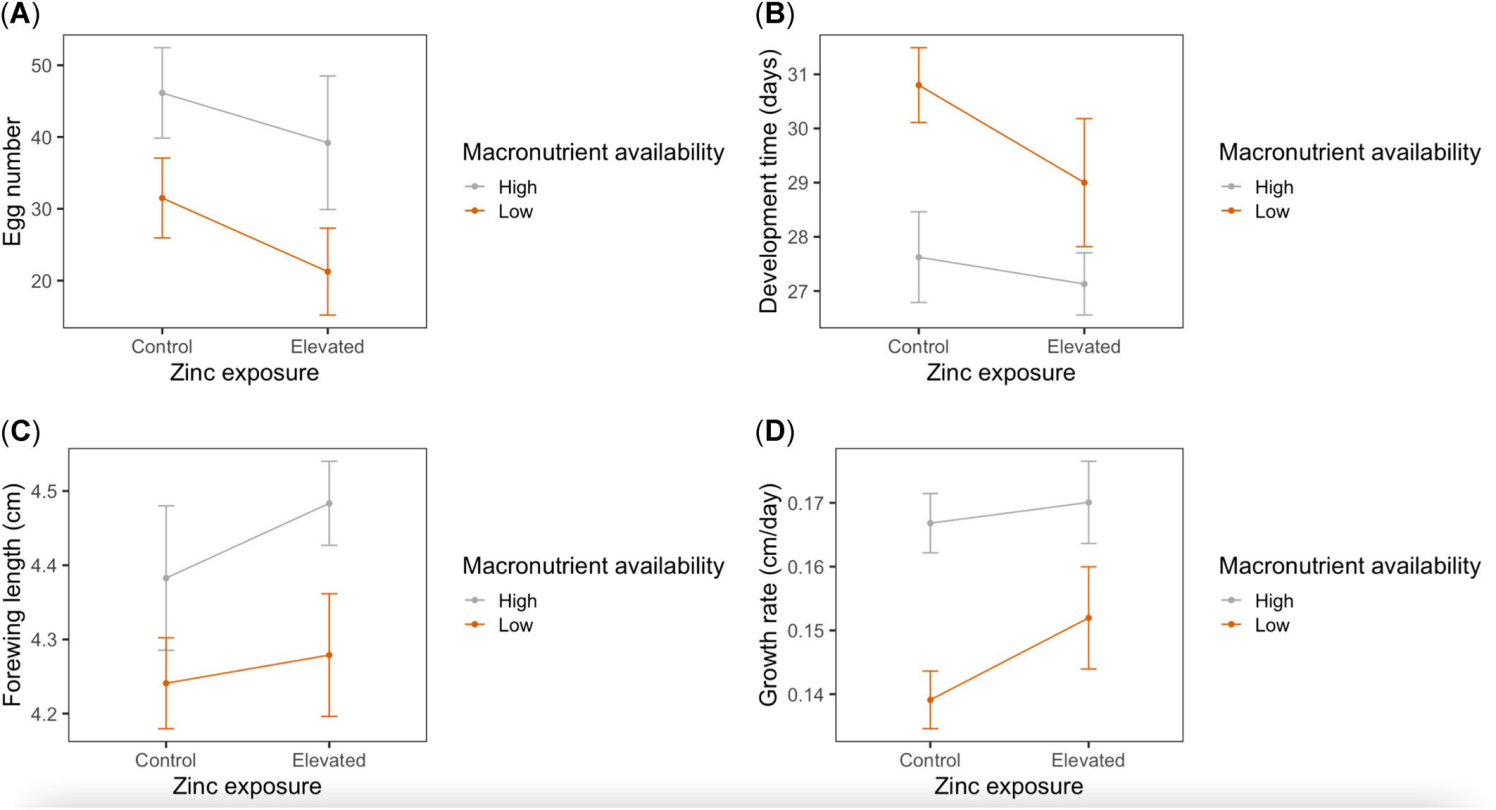
Effects of larval dietary zinc exposure and macronutrient availability on fitness‐related traits in the monarch (*Danaus plexippus*). Egg number (**A**) was measured as the total number of mature eggs dissected from *n* = 20 female ovaries at 7 days after adult emergence. Development time (**B**) of *n* = 69 larvae was measured as total number of days from egg collection to adult emergence. Forewing length (**C**) of *n* = 51 adult butterflies was measured as the distance between the forewing apex and its articulation with the thorax. Growth rate (**D**) of *n* = 51 butterflies was calculated as forewing length divided by development time. Bars indicate standard error.

There was a significant effect of macronutrient availability on egg production (*F*_1,__16_ = 5.20, *p* = 0.04; Figure 2A): females that developed on the low‐macronutrient diet had approximately 16.9 fewer mature eggs than females on the high‐macronutrient diet, on average (*t* = 2.32, *p* = 0.03). No effects of zinc exposure (*F*_1,__16_ = 1.28, *p* = 0.3) or an interaction between macronutrient availability and zinc exposure (*F*_1,__16_ = 0.05, *p* = 0.8) on egg production were detected. In addition, we detected no effects of macronutrient availability (*F*_1,__16_ = 2.00, *p* = 0.2), zinc exposure (*F*_1,__16_ = 0.11, *p* = 0.7), or an interaction between macronutrient availability and zinc exposure (*F*_1,__16_ = 0.13, *p* = 0.7) on average size of eggs produced by adult females. Across all females, forewing length was not significantly correlated with either egg number (Spearman's *ρ* = −0.02, *p* = 0.9) or egg area (Spearman's *ρ* = −0.16, *p* = 0.5), so it was not included as a covariate in either analysis.

There were significant effects of macronutrient availability on larval development time, adult forewing length, and larval growth rate (Table 3 and Figure 2B–D). Relative to individuals on the high‐macronutrient diet, individuals on the low‐macronutrient diet took approximately 2.8 days longer to develop (*t* = 3.61, *p* < 0.001), had adult forewing lengths that were approximately 4% shorter (*t* = −2.66, *p* = 0.01), and had approximately 15% slower growth rates (*t* = −4.24, *p* < 0.001), on average. There were no effects of zinc exposure or an interaction between macronutrient availability and zinc exposure on development time, forewing length, or growth rate (Table 3).

**Table 3:** Linear model results for effects of larval macronutrinet availability (high or low), zinc exposure (control or elevated), the interaction between macronutrient availability and zinc exposure, and sex on fitness‐related traits and adult thorax zinc concentration in monarchs (*Danaus plexippus*)

Trait (no.)	Macronutrient availability	Zinc exposure	Sex	Macronutrient availability × Zinc exposure
Development time (69)	*F*_1,__64_ = 13.70	*F*_1,__64_ = 2.28	*F*_1,__64_ = 0.53	*F*_1,__64_ = 0.56
	*p* < 0.001	*p* = 0.13	*p* = 0.47	*p* = 0.56
Body size (51)	*F*_1,__46_ = 6.13	*F*_1,__46_ = 0.84	*F*_1,__46_ = 0.59	*F*_1,__46_ = 0.49
	*p* = 0.02	*p* = 0.36	*p* = 0.59	*p* = 0.49
Growth rate (51)	*F*_1,__46_ = 17.84	*F*_1,__46_ = 1.66	*F*_1,__46_ = 0.47	*F*_1,__46_ = 0.53
	*p* < 0.001	*p* = 0.20	*p* = 0.47	*p* = 0.54

### Effects of zinc exposure and macronutrient availability on monarch antioxidant gene expression

**Figure 3:**
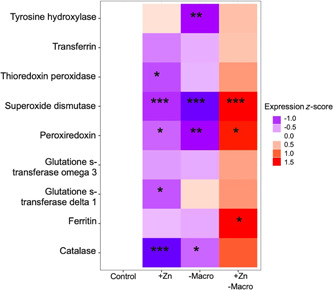
Effects of elevated zinc exposure (+Zn), low macronutrient availability (–Macro), and the combination of the two (+Zn with –Macro) on antioxidant gene expression in fifth instar monarch butterfly larvae (*Danaus plexippus*). Gene expression estimates were calculated from linear mixed effects models controlling for actin expression (fixed effect) and technical quantitative polymerase chain reaction replicate (random effect). Shaded squares indicate expression levels of nine candidate antioxidant genes relative to the control treatment (high macronutrient availability with control zinc exposure). Red shading indicates gene up‐regulation, and blue shading represents gene down‐regulation. Expression values are displayed as *z*‐scores to facilitate comparison across candidate genes.

Relative to monarchs in the high‐macronutrient/control zinc treatment (i.e., the control treatment), monarchs in the low‐macronutrient/control zinc treatment tended to have lower antioxidant gene expression (Figure 3), and expression was significantly lower for tyrosine hydroxylase, superoxide dismutase, peroxiredoxin, and catalase (Table 4). Similarly, monarchs in the high‐macronutrient/elevated zinc treatment tended to have lower antioxidant gene expression than monarchs in the control treatment (Figure 3), and expression was significantly lower for five genes: thioredoxin peroxidase, superoxide dismutase, peroxiredoxin, glutathione s‐transferase delta 1, and catalase (Table 4). In contrast, monarchs in the low‐macronutrinet/elevated zinc treatment tended to have higher antioxidant expression relative to monarchs in the control treatment (Figure 3), and expression was significantly higher for superoxide dismutase, peroxiredoxin, and ferritin; there was also a tendency for catalase expression to increase (Table 4).

**Table 4:** Linear mixed effects model results for effects of macronutrient availability, zinc exposure, and the interaction between macronutrient availability and zinc exposure on antioxidant gene expression in fat body tissue of monarch butterfly larvae (*Danaus plexippus*)^a^

Random effects			Fixed effects				
	Variance	SD		Estimate	SE	*t*	*p*
Tyrosine hydroxylase							
Replicate	<0.001	<0.001	(Intercept)	−21.79	1.83	−11.91	<0.001
			**Low‐macronutrient availability**	**−1.76**	**0.60**	**−2.94**	**0.006**
			Elevated zinc	0.35	0.60	0.59	0.56
			Low‐macronutrient availability × elevated zinc	0.73	0.86	0.86	0.40
			Actin	0.31	0.11	2.72	0.01
Transferrin							
Replicate	<0.001	<0.001	(Intercept)	−19.38	1.45	−13.33	<0.001
			Low‐macronutrient availability	−0.51	0.48	−1.08	0.29
			Elevated zinc	−0.77	0.48	−1.62	0.11
			Low‐macronutrient availability × elevated zinc	0.49	0.68	0.72	0.48
			Actin	−0.20	0.09	−2.13	0.04
Thioredoxin peroxidase							
Replicate	<0.001	<0.001	(Intercept)	−18.75	0.90	−20.87	<0.001
			Low‐macronutrient availability	−0.30	0.29	−1.03	0.31
			**Elevated zinc**	**−0.67**	**0.29**	**−2.26**	**0.03**
			Low‐macronutrient availability × elevated zinc	0.52	0.42	1.24	0.22
			Actin	0.17	0.06	3.00	0.005
Superoxide dismutase							
Replicate	<0.001	<0.001	(Intercept)	−14.23	0.71	−19.90	<0.001
			**Low‐macronutrient availability**	**−1.24**	**0.23**	**−5.31**	**<0.001**
			**Elevated zinc**	**−0.90**	**0.23**	**−3.82**	**<0.001**
			**Low‐macronutrient availability** × **elevated zinc**	**1.34**	**0.33**	**4.02**	**<0.001**
			Actin	0.32	0.05	7.09	<0.001
Peroxiredoxin							
Replicate	<0.001	0.001	(Intercept)	−13.26	0.83	−15.89	<0.001
			**Low‐macronutrient availability**	**−0.80**	**0.27**	**−2.94**	**0.006**
			**Elevated zinc**	**−0.57**	**0.27**	**−2.08**	**0.04**
			**Low‐macronutrient availability** × **elevated zinc**	**0.85**	**0.39**	**2.18**	**0.04**
			Actin	0.16	0.05	2.99	0.005
Glutathione s‐transferase omega 3							
Replicate	<0.001	<0.001	(Intercept)	−17.89	0.87	−20.47	<0.001
			Low‐macronutrient availability	−0.34	0.29	−1.17	0.25
			Elevated zinc	−0.39	0.29	−1.36	0.18
			Low‐macronutrient availability × elevated zinc	0.47	0.41	1.16	0.25
			Actin	0.19	0.05	3.42	0.001
Glutathione s‐transferase delta 1							
Replicate	<0.001	<0.001	(Intercept)	−28.24	2.13	−13.25	<0.001
			Low‐macronutrient availability	0.47	0.70	0.67	0.51
			**Elevated zinc**	**−1.52**	**0.70**	**−2.17**	**0.04**
			Low‐macronutrient availability × elevated zinc	1.26	1.00	1.26	0.22
			Actin	0.22	0.13	1.61	0.12
Ferritin							
Replicate	<0.001	<0.001	(Intercept)	−24.89	1.26	−19.70	<0.001
			Low‐macronutrient availability	−0.50	0.41	−1.21	0.23
			Elevated zinc	−0.41	0.41	−0.97	0.33
			**Low‐macronutrient availability** × **elevated zinc**	**1.37**	**0.59**	**2.33**	**0.03**
			Actin	0.19	0.08	2.41	0.02
Catalase							
Replicate	<0.001	<0.001	(Intercept)	−12.91	0.97	**−**13.28	<0.001
			**Low‐macronutrient availability**	**−0.67**	**0.32**	**−2.10**	**0.04**
			**Elevated zinc**	**−1.18**	**0.32**	**−3.72**	**<0.001**
			**Low‐macronutrient availability** × **elevated zinc**	**0.86**	**0.45**	**1.89**	**0.06**
			Actin	0.15	0.06	2.51	0.02

^a^ Each model controls for variation in actin expression (fixed effect) and technical quantitative polymerase chain reaction replicate (random effect). Bold type indicates model effects that are statistically significant (p < 0.05).

## DISCUSSION

Consistent with our initial hypothesis that tolerance to heavy metal contamination is macronutrient dependent, we found that elevated zinc exposure negatively impacted survival only of monarchs developing on a low‐macronutrient diet (Figure 1). Given the expectation that heavy metal tolerance is physiologically costly (Morgan et al., 2007; Posthuma & Van Straalen, 1993), the underlying costs associated with maintenance or production of tolerance mechanisms are likely greater for individuals with fewer nutritional resources. Indeed, monarchs developing on the low‐macronutrient diet had prolonged development time, smaller adult body size, slower growth rate, and reduced egg production (Figure 2), suggesting that they were more energy limited. Our results support the more general view that variation in fitness costs depends on resource or nutrient availability, a concept often invoked to predict variation in life‐history tradeoffs (Bonduriansky, 2007; Cody, 1966; Houle, 1991; van Noordwijk & de Jong, 1986; Zera & Harshman, 2001).

In nature, resource dependence may help explain why individuals vary in their abilities to tolerate pollutants across spatial scales. For instance, nutrient availability may vary greatly across landscapes, either naturally or due to differential nutrient inputs from human activities (e.g., nitrogen or phosphorous from agriculture; Vitousek et al., 1997). Anthropogenic increases in nutrient availability affect organismal allocation to fitness components (Jeyasingh & Weider, 2005; Morehouse & Rutowski, 2010; Sterner, 1993), and in this manner, fitness costs associated with pollution tolerance might be lower for organisms developing in more nutrient‐rich patches. Although our study considers how organismal pollution tolerance is impacted by variation in dietary nutrient availability, there may still be significant genetic variation within populations for the ability to acquire or assimilate nutrients from the environment. Such variation could yield more complex genotype‐by‐environment interactions for metal tolerance across nutritionally varying patches (Snell‐Rood et al., 2015). Future work quantifying genetic variation for tolerance across different levels of nutrient availability may provide more detailed insights into metapopulation dynamics and spatial sorting of genotypes across polluted landscapes.

Monarchs developing on the low‐macronutrient diet with elevated zinc pollution had higher antioxidant gene expression than monarchs developing on the control diet (i.e., high‐macronutrient diet with no added zinc pollution; Figure 3). Given that organisms experiencing oxidative stress are expected to respond by up‐regulating antioxidant pathways (De Block & Stoks, 2008; Krishnan & Kodrík, 2006; Mittapalli et al., 2007), this result is consistent with the idea that monarchs with lower access to nutritional resources faced greater oxidative stress burdens under metal exposure. Although these individuals tended to increase expression of all antioxidant genes considered, we found significant up‐regulation of genes specifically involved in defense against ROS (Table 4): superoxide dismutase, catalase, and peroxiredoxin. We also found significant up‐regulation of ferritin, which contributes to metal storage and transport. In contrast, we detected no significant up‐regulation of tyrosine hydroxylase, the enzyme that synthesizes melanin. Although melanin may contribute to heavy metal detoxification in vertebrates (Goiran et al., 2017), melanin has diverse functions in insects (e.g., immunity, ornamentation, cuticle hardening, and thermoregulation; Stoehr, 2006), which might constrain its role in heavy metal tolerance. In addition, neither of the glutathione genes considered (glutathione s‐transferase delta 1 or glutathione s‐transferase omega 3) were significantly up‐regulated, even though glutathione s‐transferase omega 3 has previously been implicated in antioxidant defenses against heavy metals in insects (Lee et al., 2015). Given that glutathiones and other detoxification pathways are more costly to produce than antioxidant enzymes such as superoxide dismutase and catalase (Gems & Partridge, 2013; Speakman & Garratt, 2014), it is possible that glutathione expression is more constrained under conditions of low nutrient availability. Further support for the idea that antioxidant expression may have energetic costs comes from our finding that monarchs developing on the low‐macronutrient diet in the absence of zinc pollution had generally lower levels of antioxidant expression relative to monarchs on the control treatment (Figure 3 and Table 4). This suggests that nutrient‐limited individuals might reduce antioxidant expression to conserve energy in otherwise benign environments but still retain the ability to up‐regulate these responses if the environment becomes stressful. Additional support for this hypothesis comes from a previous study in which *Lestes viridis* larvae displayed lower levels of superoxide dismutase and catalase during a period of food limitation, but dramatically increased production of these antioxidants during subsequent oxidative stress induced by compensatory growth (De Block & Stoks, 2008).

Although zinc pollution increased antioxidant expression in monarchs developing on the low‐macronutrient diet, monarchs exposed to zinc pollution on the high‐macronutrient diet displayed significantly lower expression of several antioxidant genes (superoxide dismutase, peroxiredoxin, catalase, glutathione s‐transferase delta 1, and tyrosine hydroxylase) compared with monarchs on the control treatment (Table 4 and Figure 3). This is inconsistent with our prediction that elevated zinc exposure would generally increase antioxidant expression, regardless of nutrient availability. Why would zinc have opposite effects on antioxidant gene expression in individuals with high and low access to macronutrients? One possibility is that responses to zinc might differ depending on whether zinc is having overall toxic or nutritional effects on the organism. As a toxicant, zinc generates harmful ROS by interfering with enzyme activities and mitochondrial function (Capasso et al., 2005; Gazaryan et al., 2007). As a nutrient, zinc has the opposite effect by serving as a powerful antioxidant that can protect molecules from ROS damage and regulate cellular metal homeostasis (Formigari et al., 2007; Powell, 2000). Our results are consistent with the idea that zinc acted as a toxicant in individuals with lower access to macronutrient resources: monarchs exposed to zinc on the low‐macronutrient diet had lower survival and higher expression of antioxidant genes (Figures 1 and 3), suggesting that they were responding to increased oxidative stress burdens. However, in monarchs developing on the high‐macronutrient diet, there was no evidence that zinc negatively impacted performance (Figure 2), and survival even tended to increase (Figure 1). The fact that these individuals also had lower expression of antioxidant genes (Figure 3) suggests that zinc had much different effects on their oxidative physiology, which could potentially be related to the nutritional and regulatory properties of zinc.

It is unclear why a given level of zinc exposure might have harmful effects in individuals with low access to macronutrient resources but potential nutritional and regulatory benefits in individuals with high access to macronutrient resources. One hypothesis is that more nutrient‐limited individuals have fewer energetic resources available to invest in physiological machinery required to efficiently regulate internal zinc levels. For instance, key regulators of zinc homeostasis are the metallothionein proteins, which sequester zinc, enable its antioxidant properties, and play a central role in detoxification (Capasso et al., 2005; Gazaryan et al., 2007; Klaassen et al., 1999). Such regulatory systems may be energetically costly (Barber et al., 1990; Forbes & Calow, 1996), because sublethal heavy metal exposures have been shown to increase organismal metabolic rates by 32%–175% (Hopkins et al., 1999; Rowe, 1998; Rowe, Kinney, et al., 1998). Given these potential energetic constraints, nutrient‐limited individuals may struggle more to effectively regulate internal zinc loads relative to individuals with higher access to resources. The inability to effectively regulate zinc may increase oxidative stress levels and damage to organ structures (Shu et al., 2012). In contrast, high‐condition individuals may better manage physiological costs of zinc regulation, preventing zinc‐induced damage and perhaps even benefiting from the regulatory properties of zinc.

In summary, by showing that monarch survival and underlying antioxidant responses to zinc contamination are influenced by dietary macronutrient availability, our study supports the hypothesis that organismal tolerance to anthropogenic pollution depends on the availability of dietary nutrients. Our results suggest that considering interactions between stressors can broaden conservation insights and draw attention to the possibility that laboratory studies may underestimate impacts of anthropogenic pollutants on wild populations if effects of naturally occurring stressors (e.g., nutrient or resource fluctuations) are not also considered (Sih et al., 2004). Our results are consistent with recent ecotoxicological studies showing that reduced access to major dietary macronutrients (e.g., phosphorous) can lower organismal tolerance of toxic chemicals such as pesticides (Ieromina et al., 2014) and nonessential heavy metals (Arce‐Funck et al., 2016). However, as just discussed, our results suggest that zinc not only was harmful under conditions of low‐macronutrient availability but might have been beneficial in the context of high‐macronutrient availability. These results suggest the hypothesis that variation in macronutrient availability might have a key influence on whether contaminants such as zinc are overall detrimental or beneficial to organismal performance. Future studies can test this hypothesis using ecological stoichiometric approaches that carefully manipulate dietary ratios of contaminants and specific nutrients (e.g., nitrogen, phosphorous, or potassium; see Peace et al., 2021).

## Supporting Information

The Supporting Information is available on the Wiley Online Library at https://doi.org/10.1002/etc.5305.

## Author Contributions Statement

**Alexander M. Shephard**: Conceptualization; methodology; formal analysis; funding acquisition; investigation; visualization; writing—original draft. **Emilie C. Snell‐Rood**: Conceptualization; funding acquisition; methodology; writing—editorial advice. **Noah S. Brown**: Investigation; writing—editorial advice.

## Supporting information

This article includes online‐only Supporting information.

Figure S1.

Supplementary information.

Supplementary information.

## Data Availability

Data, associated metadata, and calculation tools are also available from the corresponding author (sheph095@umn.edu).
