# Supplementary figures and images for: Anthropogenic Zinc Exposure Increases Mortality and Antioxidant Gene Expression in Monarch Butterflies with Low Access to Dietary Macronutrients
Source: Environ Toxicol Chem. 2022 Mar 14;41(5):1286–96. doi: 10.1002/etc.5305 (PMC9314993; doi:10.1002/etc.5305)

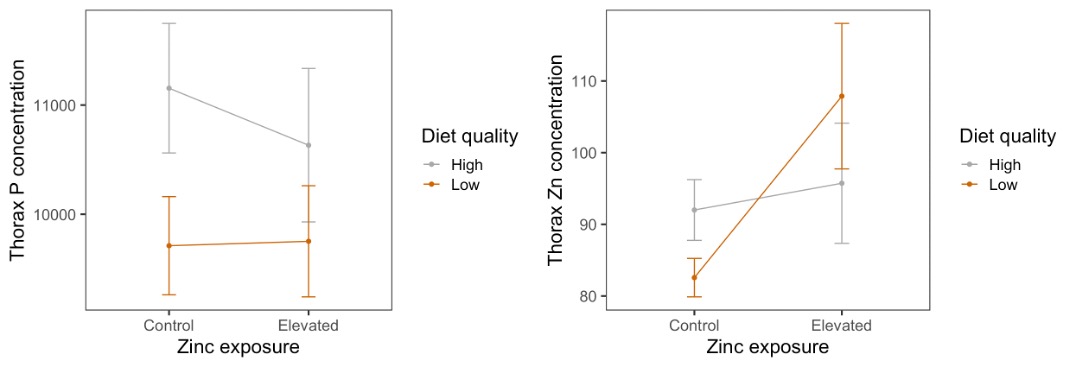

Supplement: Supplementary file 1 — Figure S1. [file ETC-41-1286-s003.jpg]
