# Supplementary material for: Anthropogenic Zinc Exposure Increases Mortality and Antioxidant Gene Expression in Monarch Butterflies with Low Access to Dietary Macronutrients
Source: Environ Toxicol Chem. 2022 Mar 14;41(5):1286–96. doi: 10.1002/etc.5305 (PMC9314993; doi:10.1002/etc.5305)
Supplement: Supplementary file 2 — Supplementary information. [file ETC-41-1286-s002.docx]

**Table S1.** Linear model results for effects of larval macronutrient availability (high or low), zinc exposure (control or elevated), the interaction between macronutrient availability and zinc exposure, and sex on adult thorax zinc and phosphorous concentrations (mg/kg) in the monarch butterfly (*D. plexippus*).

| **Trait (*N*)** | **Macronutrient availability** | **Zinc exposure** | **Sex** | **Macronutrient availability x Zinc exposure** |
| --- | --- | --- | --- | --- |
| Adult thorax zinc concentration (24) | F_1, 19_ = 0.036  P = 0.85 | F_1, 19_ = 4.09  P = 0.057 | F_1, 19_ = 0.045  P = 0.83 | F_1, 19_ = 2.48  P = 0.13 |
| Adult thorax phosphorous concentration (24) | F_1, 19_ = 4.31  P = 0.052 | F_1, 19_ = 0.18  P = 0.67 | F_1, 19_ = 1.59  P = 0.22 | F_1, 19_ = 0.58  P = 0.46 |
