# Supplementary material for: Anthropogenic Zinc Exposure Increases Mortality and Antioxidant Gene Expression in Monarch Butterflies with Low Access to Dietary Macronutrients
Source: Environ Toxicol Chem. 2022 Mar 14;41(5):1286–96. doi: 10.1002/etc.5305 (PMC9314993; doi:10.1002/etc.5305)
Supplement: Supplementary file 3 — Supplementary information. [file ETC-41-1286-s001.docx]

**Table S2.** Linear mixed effects model results for effects of larval macronutrient availability, zinc exposure, and the interaction between the two on expression of two candidate normalizing genes for qPCR analyses in monarch butterfly larvae (*Danaus plexippus*): actin and elongation factor 1-alpha. In each model, technical qPCR replicate is included as a random effect.

| **Random effects** | | | **Fixed effects** | | | | |
| --- | --- | --- | --- | --- | --- | --- | --- |
|  | **Variance** | ***SD*** |  | **Estimate** | ***SE*** | ***t*** | ***p*** |
| Actin | | | | | | | |
| replicate | <0.001 | <0.001 | (Intercept) | -15.36 | 0.61 | -25.20 | <0.001 |
|  |  |  | Low macronutrient availability | 0.35 | 0.86 | 0.41 | 0.67 |
|  |  |  | Elevated zinc | 0.24 | 0.86 | 0.28 | 0.78 |
|  |  |  | Low macronutrient availability x Elevated zinc | 1.10 | 1.22 | 0.90 | 0.37 |
| Elongation factor 1-alpha | | | | | | | |
| replicate | <0.001 | <0.001 | (Intercept) | -12.92 | 0.21 | -61.12 | <0.001 |
|  |  |  | **Low macronutrient availability** | **-0.65** | **0.30** | **-2.18** | **0.04** |
|  |  |  | Elevated zinc | -0.30 | 0.30 | -1.01 | 0.32 |
|  |  |  | **Low macronutrient availability x Elevated zinc** | **0.90** | **0.42** | **2.14** | **0.04** |
